# Association of *GLP1R* variants rs2268641 and rs6923761 with obesity and other metabolic parameters in a Polish cohort

**DOI:** 10.3389/fendo.2022.1000185

**Published:** 2022-10-19

**Authors:** Joanna Michałowska, Ewa Miller-Kasprzak, Agnieszka Seraszek-Jaros, Adrianna Mostowska, Paweł Bogdański

**Affiliations:** ^1^ Department of Treatment of Obesity, Metabolic Disorders and Clinical Dietetics, Poznan University of Medical Sciences, Poznan, Poland; ^2^ Department of Bioinformatics and Computational Biology, Poznan University of Medical Sciences, Poznan, Poland; ^3^ Department of Biochemistry and Molecular Biology, Poznan University of Medical Sciences, Poznan, Poland

**Keywords:** obesity, GLP1R gene, single nucleotide variant, metabolic syndrome, GLP-1, metabolic health

## Abstract

**Introduction:**

Obesity is a complex disease associated with excessive fat accumulation and numerous metabolic complications. So far, many factors leading to the development of this disorder have been identified, including genetic susceptibility. Various studies linked *GLP1R* variants with anthropometric and metabolic parameters, suggesting the role of the variation in this gene in metabolic health.

**Objective:**

The aim of this study is to investigate the association of two single nucleotide variants of *GLP1R* gene, rs2268641 and rs6923761, with excessive weight, metabolic syndrome, anthropometric measurements and selected metabolic parameters.

**Methods:**

Normal-weight subjects (n= 340, control group) and subjects with excessive body mass (n = 600, study group) participated in this study. For all participants, anthropometric measurements and metabolic parameters were collected, and genotyping of the two single nucleotide variants of *GLP1R* gene, rs2268641 and rs6923761, was performed using the high-resolution melting curve analysis.

**Results:**

Significant differences in the genotype distribution of rs2268641 were found, where homozygous TT genotype was significantly less frequent in the study group with excessive body mass (OR=0.66; p=0.0298). For rs6923761, A allele and homozygous AA genotype were significantly more frequent in the study group with excessive weight than in the control group (OR=1.27; p=0.0239 and OR=1.69; p=0.0205, respectively). The association of studied variants with metabolic parameters was found for rs6923761. For this variant, AA carriers had higher body mass in comparison to GG carriers (p=0.0246), and AA carriers had higher glucose concentration in comparison to AG carriers (p=0.0498). We did not find an association of rs2268641 and rs6923761 with metabolic syndrome.

**Conclusion:**

In our study, AA carriers of rs6923761 had higher risk of excessive body mass, whereas TT carriers of rs2268641 had lower risk of being overweight. Moreover, homozygous carriers of the minor allele of rs6923761 had higher glucose concentration in comparison to heterozygous subjects. None of the studied variants were associated with metabolic syndrome in the studied population.

## Introduction

Obesity can be defined as an excessive fat accumulation that significantly impairs overall health (1–3). The prevalence of this disease has been rising in the past 50 years, with approximately one-third of the worldwide population currently being obese or overweight. However, absolute prevalence rates differ and depend on the country, region and ethnicity (1). Obesity is a complex chronic disease, which is associated with numerous comorbidities. These include, but are not limited to, metabolic syndrome (MetS), type 2 diabetes (T2D), cardiovascular disease (CVD), hypertension, and nonalcoholic fatty liver disease (NAFLD) (4). The fundamental cause of obesity is a long-term positive energy balance; however, there are various mechanisms leading to the development of this condition. Overconsumption of calories due to the excessive intake of high energy-dense and palatable foods combined with a sedentary lifestyle largely explain energy imbalance and higher risk of excessive weight gain. Beyond diet, other components contribute to the risk of obesity, including environmental, behavioral, developmental and genetic factors (5). So far, genome-wide association studies (GWAS) have identified more than 1000 genetic loci associated with adiposity outcomes (6). Most of these were determined in subjects of European ancestry for body mass index (BMI), a proxy for overall body adiposity, or BMI-adjusted waist-to-hip ratio (WHR), which is a proxy for body fat distribution (6, 7). *FTO* (FTO alpha-ketoglutarate dependent dioxygenase; HGNC:24678) was the first gene associated with obesity identified by GWAS (8). A minor allele of rs1558902 variant of this gene was associated with BMI increase by 0.39 kg/m^2^ and a 1.2-fold increase in obesity risk based on the data from 247,796 individuals of European ancestry (9). Nonetheless, apart from several rare cases associated with monogenic obesity, it is unlikely that genetic factors can be an underlying cause of excessive weight (5). Most often, obesity results from complex interactions among multiple genes and environmental factors (multifactorial obesity). Therefore, some individuals living in an obesity-prone environment might be more susceptible to weight gain than others (10).

*GLP1R* (Glucagon-Like Peptide 1 Receptor; HGNC:4324) is one of the genes associated with obesity and T2D risk identified by GWAS (11, 12). The *GLP1R* gene encodes a 7-transmembrane protein, a receptor for glucagon-like peptide 1 (GLP-1) hormone (13). This hormone exerts insulinotropic activity and affects glucagon secretion as glucagon-like peptide 1 receptor (GLP-1R) is predominantly localized in β- and α-cells of the pancreas (14). Except for the pancreas, the research confirmed the expression of this receptor in the central and peripheral nervous system, gastrointestinal tract, cardiovascular system, kidney, and lung. Therefore, beyond the pancreatic effect, GLP-1 exhibits other functions, including the regulation of food intake, appetite, lipid metabolism, body weight, energy expenditure, and the cardiovascular system (15).

Research showed that *GLP1R* single nucleotide variants (SNVs) rs2268641 and rs6923761 are associated with obesity and anthropometric measurements. Rs2268641 (C>T) is located on chromosome 6 in position 39082490 with the Minor Allele Frequency (MAF) 0.349 (1000 Genomes; European population). The transcript variant occurs within an intron, and it has been classified as benign according to the American College of Medical Genetics (ACMG) classification. Rs6923761 (G>A/C) is a missense variant located on chromosome 6 in position 39066296 with MAF for A allele equal to 0.105 (1000Genomes, European population). Minor A allele is associated with glycine to serine substitution (Gly168Ser). Rs6923761 variant was also classified as benign according to ACMG classification. De Luis et al. found that A allele carriers of rs6923761 were characterized by lower body mass and better anthropometric parameters, i.e. BMI, waist circumference (WC), fat mass and waist to hip ratio (16–22). This gene variant was also associated with various metabolic parameters, including blood pressure (BP), cholesterol, glucose, insulin, triglycerides (TG) and c-reactive protein (CRP) (16–22). All performed studies were carried out on obese subjects with or without T2D. Normal-weight subjects were not included in any of the analyses. Moreover, genetic association analysis of 30 genes related to obesity using a Bayesian hierarchical generalized linear model found that the rs2268641 gene variant of *GLP1R* was significantly associated with BMI (11). To our knowledge, this is the only study linking the rs2268641 variant with excessive weight, and other research investigating the relationship between this SNV and anthropometric or metabolic parameters is not available.

To address the research gap in this field, the objective of the study is to establish the association of two *GLP1R* SNVs, rs2268641 and rs6923761, with excessive body mass, MetS, anthropometric measurements and selected metabolic parameters in the Polish population.

## Materials and methods

### Population

The study was approved by the Ethics Committee at Poznan University of Medical Sciences (approval no. 643/20). All participants received oral and written information about the research and signed informed consent. Data of 940 adult (≥18 years old) subjects were included in the analysis. Participants were divided into two groups according to the BMI – control group (n = 340, BMI <25 kg/m^2^) and study group with excessive body mass (n = 600, BMI ≥25 kg/m^2^). There were 76 diabetic subjects (13%) in the study group, whereas 16 normal-weight subjects (5%) in the control group had T2D. To investigate the relationship of SNVs rs2268641 and rs6923761 with MetS, the presence of its’ criteria was determined for all subjects.

### Procedures

Anthropometric measurements and blood sample collection was performed for all participants. Anthropometric measurements included body weight, height, and waist and neck circumference. During the assessment, participants wore light clothes and no shoes to minimize the measurement error. Body mass was measured to the nearest 0.1 kg, and height was measured to the nearest 1 cm using electronic scales with a stadiometer (Charder MS4900). BMI was calculated by dividing the body mass [kg] by the square of the body height [m]. According to this index, normal weight is defined as BMI higher or equal to 18.5 kg/m^2^ to 24.9 kg/m^2^, overweight is defined as BMI higher or equal to 25 kg/m^2^ to 29.9 kg/m^2^, and obesity is defined as BMI≥30 kg/m^2^ (23). WC was measured at the middle point between the iliac crest and the lowest rib. Neck circumference (NC) was measured at a point just below the larynx and perpendicularly to the long axis of the neck. WC and NC measurements were performed using a certified tape measure (Seca 201).

The presence of MetS was determined using criteria presented in the joint interim statement of the International Diabetes Federation (IDF); National Heart, Lung, and Blood Institute (NHLBI); American Heart Association (AHA); World Heart Federation (WHF); International Atherosclerosis Society (IAS); and International Association for the Study of Obesity (IASO). According to this statement, MetS can be recognized if at least three of the following five medical conditions are present: WC ≥94 cm for males or ≥80 cm for females; TG ≥150 mg/dl (or treatment for elevated TG); high-density lipoprotein (HDL) ≤40 mg/dl for male or ≤50 mg/dl for female (or treatment for reduced HDL); systolic BP ≥ 130 and/or diastolic BP ≥ 85 mm Hg (or antihypertensive treatment); and fasting glucose ≥ 100 mg/dl (or drug treatment for elevated glucose) (24).

### Biochemical assays

Venous blood samples were collected from all participants from a cubital vein. All subjects were in the fasting state, as the collection was scheduled in the morning, 10-14 hours after the last meal. The blood samples were centrifuged and frozen at −80°C. Glucose, TG, HDL, alanine transaminase (ALT) and aspartate transaminase (AST) were measured using standardized commercial tests.

### Genotyping

Genomic DNA was isolated from peripheral blood lymphocytes through the salting out technique. Genotyping of the *GLP1R* SNVs rs2268641 and rs6923761 was performed using the high-resolution melting curve (HRM) analysis with 5x HOT FIREPol EvaGreen HRM Mix (Solis BioDyne, Tartu, Estonia) on the Light Cycler 96 system (Roche Diagnostics, Mannheim, Germany). For quality control, approximately 10% of the randomly chosen samples were regenotyped using the same genotyping method. The concordance rate was 100%. Samples that failed the genotyping were excluded from further statistical analysis. HRM parameters and primer sequences for analyzed SNVs are presented in **Supplementary Materials** (**Supplementary Table 1**). The HRM genotyping method was validated using Sanger sequencing. For each SNV, the Hardy-Weinberg (HW) equilibrium was evaluated in each group by the Chi-square (χ2) test. Statistically significant deviation from HW expectations was interpreted as p-value <0.05. Linkage disequilibrium (LD) and haplotype association analyses were performed using Haploview 4.2 software.

### Statistical analysis

All calculations and statistical analyses were performed using Statistica (data analysis software system) version 13 (Tulsa, OK, USA) TIBCO Software Inc (2017). Quantitative data are presented using mean ± standard deviation (SD), and the compatibility of their distribution with the Gaussian curve was checked using the Shapiro-Wilk test. Since the test confirmed a lack of normality, non-parametric methods were used for statistical analysis. Differences between the control group and the study group were verified by the Mann-Whitney test. Differences between genotypes were verified by the Kruskal-Wallis test and the *post-hoc* test when a statistically significant difference was found. Categorial variables are presented using frequency and percentages. Deviations from Hardy-Weinberg equilibrium expectations were determined using the χ2 test. The same test was used to compare groups. Odds ratios (ORs) and 95% confidence intervals (CIs) were calculated. Additionally, in MedCalc^®^ Statistical Software version 20.027 (MedCalc Software Ltd, Ostend, Belgium), the logistic regression was performed to investigate if any of the studied SNVs is associated with elevated or lower risk of excessive weight or MetS. For all tests, results were considered significant if the p-value was less than 0.05.

## Results

Data of 940 subjects were included in the analysis. Based on the BMI, 340 participants were assigned to the control group (BMI <25 kg/m^2^) and 600 participants were assigned to the study group with excessive body mass (BMI ≥25 kg/m^2^). The characteristics of the study and the control group is presented in **Table 1**. The mean age in the control group was 48 years old ( ± SD 14.5) and 57 years old ( ± SD 13.2) in the study group. Mean BMI was 22.3 kg/m^2^ and 29.7 kg/m^2^ respectively. Both groups presented significant differences for all parameters. Women accounted for 61.5% (n= 369) of the study group, and 79% (n= 269) of the control group. Thirteen percent (n= 38) of the control group and 52% (n=288) of the study group met the criteria for MetS. Its presence was assessed for 851 subjects, as due to the insufficient data, it was not possible to determine MetS status for 89 participants.

**Table 1:** The characteristics of the study population.

	Control group N = 340		Study group N = 600		p-value	
	Mean	SD	Mean	SD		
Age [years]	48.42	14.52	56.88	13.19	< 0.0001	
Body weight [kg]	62.15	8.72	82.29	13.32	< 0.0001	
BMI [kg/m^2^]	22.33	1.79	29.72	4.06	< 0.0001	
WC [cm]	79.76	9.28	99.42	11.97	< 0.0001	
NC [cm]	33.81	3.03	37.62	3.52	< 0.0001	
Glucose [mg/dl]	90.20	16.57	98.40	27.90	< 0.0001	
TG [mg/dl]	111.90	69.12	170.39	121.50	< 0.0001	
HDL [mg/dl]	73.15	17.96	60.55	15.36	< 0.0001	
AST [IU/l]	26.26	7.64	29.48	12.97	< 0.0001	
ALT [IU/l]	24.33	11.63	34.97	23.59	< 0.0001	
**Sex and metabolic syndrome status**					
	N	%	N	%		
Sex					
*women*	269	79	369	61.5	< 0.0001	
*men*	71	21	231	38.8		
Metabolic syndrome					
*no*	256	87	269	48	< 0.0001	
*yes*	38	13	288	52		

SD, standard deviation; BMI, Body Mass Index; WC, waist circumference; NC, neck circumference; TG, triglycerides; HDL, high density lipoprotein; AST, aspartate transaminase; ALT, alanine transaminase. The differences between the control group and the study group were verified by the Mann-Whitney test. Control group included participants with BMI <25 kg/m^2^ and subjects with BMI ≥25 kg/m^2^ were assigned to the study group.

Studied SNVs were in low LD (r^2^ = 0.225). For each SNV, there was no statistically significant deviation from the Hardy–Weinberg equilibrium for both the control and the study group. The following number of samples failed the genotyping: 34 samples for rs2268641 (0.04%) and 17 samples (0.02%) for rs6923761. Statistical analysis of the distribution of the particular alleles and genotypes of rs2268641 did not show any significant differences between the frequency of alleles T and C. However, there was a significant difference in the genotypes distribution, where homozygous TT genotype was significantly less frequent in the study group with excessive body mass (OR=0.66; p=0.0298). The possible role of rs6923761 as a risk factor for excessive body mass was also studied. For this SNV, G allele was more frequent in the control group. A allele and homozygous AA genotype were more frequent in the study group with excessive body mass (OR=1.27, p=0.0239 and OR=1.69, p=0.0205; respectively). The frequencies of alleles and genotypes of *GLP1R* variants in the study and the control group are presented in **Table 2**.

**Table 2:** The frequencies of *GLP1R* variants in the study and the control group.

*GLP1R* variant	Study group% (N)	Control group% (N)	OR (95% CI)	p-value
**rs2268641**				
*allele*				
*T*	40	42	0.90 (0.74-1.09)	0.2955
*C*	60	58	1.11 (0.92-1.35)	
*genotypes*				
*TT*	14.0 (81)	19.6 (64)	0.66 (0.46-0.95)	0.0298
*CT*	51.5 (299)	45.4 (148)	1.28 (0.97-1.68)	0.0835
*CC*	34.5 (200)	35.0 (114)	0.97 (0.74-1.30)	0.8846
*p*HW	0.0659	0.2053		
**rs6923761**				
*allele*				
*A*	35	30	1.27 (1.04-1.56)	0.0239
*G*	65	70	0.79 (0.64-0.97)	
*genotypes*				
*AA*	14.0 (82)	8.7 (29)	1.69 (1.08-2.65)	0.0205
*AG*	43.0 (254)	42.9 (143)	1.00 (0.77-1.32)	1.0000
*GG*	43.0 (254)	48.4 (161)	0.81 (0.62-1.06)	0.1297
*p*HW	0.1518	0.7292		

N, number of participants; OR, odds ratio; CI, confidence interval; pHW, Hardy-Weinberg p-value. Analysis performed using the χ2 test.

Statistical analysis of the frequencies of the particular alleles and genotypes of rs2268641 and rs6923761 in subjects with or without MetS did not show statistically significant differences. For each SNV, there was no statistically significant deviation from the Hardy–Weinberg equilibrium for both groups. The distribution of particular alleles and genotypes of rs2268641 and rs6923761 in subjects with or without MetS is presented in **Table 3**.

**Table 3:** The frequencies of *GLP1R* variants in the group with and without metabolic syndrome.

*GLP1R* variants	With MetS% (N)	Without MetS% (N)	OR (95% CI)	p-value
**rs2268641**				
*allele*				
*T*	41	41	0.97 (0.79-1.19)	0.7981
*C*	59	59	1.03 (0.84-1.26)	
*genotypes*				
*TT*	16.0 (51)	16.2 (84)	0.98 (0.67-1.43)	1.0000
*CT*	49.2 (157)	50.1 (259)	0.97 (0.73-1.28)	0.8310
*CC*	34.8 (111)	33.7 (174)	1.05 (078-1.41)	0.7640
*pHW*	0.7153	0.4497		
**rs6923761**				
*allele*				
*A*	35	33	1.13 (0.92-1.39)	0.2667
*G*	65	67	0.89 (0.72-1.09)	
*genotypes*				
*AA*	13.9 (45)	10.3 (54)	1.42 (0.93-2.16)	0.1229
*AG*	42.7 (138)	44.7 (235)	0.92 (0.70-1.22)	0.6182
*GG*	43.4 (140)	45.0 (237)	0.93 (0.71-1.23)	0.6696
*pHW*	0.2457	0.7037		

MetS, Metabolic Syndrome; n, number of participants; OR, odds ratio; CI, confidence interval; pHW, Hardy-Weinberg p-value. Analysis performed using the χ2 test.

The logistic regression results showed that CT carriers of the rs2268641 variant have a 56% higher risk of having excessive body weight in comparison to TT carriers (OR=1.56; 95% CI:1.09-2.23; p=0.0141). However, the statistical significance of this association was lost after adjusting the model for sex and age. On the contrary, the GG genotype of rs6923761 was associated with a 38% lower risk of excessive body weight in comparison to AA carriers (OR=0.62; 95%CI:0.39-0.98). The association was still significant after adjusting for sex and age. Particular genotypes of studied SNVs were not associated with elevated or lower risk of MetS. The results of performed logistic regression are presented in **Table 4**.

**Table 4:** Logistic regression investigating the association of rs2268641 and rs6923761 variants with excessive weight and metabolic syndrome.

	Excessive weight^1^						Metabolic syndrome					
	OR	95% CI	p	OR	95% CI	p	OR	95% CI	p	OR	95% CI	p
rs2268641	CT*			CC*			CT*			CC*		
raw	1.56	1.09-2.23	0.0141	1.35	0.93-1.97	0.1119	1.06	0.73-1.54	0.7571	1.12	0.75-1.66	0.5864
adj.	1.38	0.94-2.02	0.0920	1.15	0.77-1.71	0.5040	0.98	0.66-1.47	0.0457	0.98	0.64-1.49	0.9158
rs6923761	AG**			GG**			AG**			GG**		
raw	0.69	0.45-1.08	0.1023	0.62	0.40-0.95	0.0290	0.78	0.51-1.18	0.2383	0.78	0.51-1.19	0.2487
adj.	0.65	0.41-1.04	0.0713	0.62	0.39-0.98	0.0418	0.71	0.45-1.11	0.1321	0.8	0.51-1.26	0.0361

OR, Odds Ratio; CI, Confidence Interval; adj., adjusted for sex and age; ^1^ BMI≥25 kg/m^2^.

*TT carriers used as a reference **AA carriers used as a reference.

To investigate if any particular genotype is associated with differences in anthropometric measurements and selected metabolic parameters, we compared the age, body mass, BMI, WC, NC, and glucose, ALT, AST, TG and HDL concentration in every genotype group. The analysis did not show any differences between TT, CT and CC carriers of rs2268641 for those parameters (**Supplementary Table 2**). For the rs6923761 variant, the analysis showed significant differences in body mass and glucose concentration, where AA carriers had higher body mass in comparison to GG carriers (mean 78.66 kg ± SD =15.64 and mean 74.1 kg ± SD=14.91 respectively; p=0.0246). Moreover, AA carriers had higher glucose concentration, in comparison to AG carriers (mean 99.49 mg/dl ± SD=25.94 and 95.37 mg/dl ± SD=27.23 respectively; p=0.0498). There were no differences between particular genotypes of rs6923761 and age, BMI, WC, NC, TG, HDL, AST and ALT. The results are presented in **Table 5**.

**Table 5:** Comparison of anthropometric measurements and selected metabolic parameters between particular genotypes of rs6923761 variant.

rs6923761	AA (N=111)		AG (N=397)		GG (N=415)		p
	Mean	SD	Mean	SD	Mean	SD	
Age [years]	54.01	14.01	54.65	14.26	52.81	14.34	0.2085
Body mass [kg]	78.66	15.64	74.98	15.42	74.10	14.91	0.0246
BMI [kg/m^2^]	27.94	5.58	27.14	4.90	26.72	4.71	0.0911
WC [cm]	94.73	14.82	92.94	14.57	91.24	14.29	0.0743
NC [cm]	36.83	4.27	36.49	3.79	36.25	3.71	0.6475
Glucose [mg/dl]	99.49	25.94	95.37	27.23	94.77	21.89	0.0498
TG [mg/dl]	162.05	124.05	146.82	101.61	148.17	113.29	0.3632
HDL [mg/dl]	64.21	15.45	64.43	17.07	65.11	17.63	0.8561
AST	27.44	6.83	28.81	12.13	28.35	12.21	0.5547
ALT	29.46	13.25	32.17	24.41	31.37	19.41	0.9299

SD, standard deviation; BMI, Body Mass Index; WC, waist circumference; NC, neck circumference; TG, triglycerides; HDL, high density lipoprotein; AST, aspartate transaminase; ALT, alanine transaminase; Kruskal-Wallis test was performed and when p<0.05 post hoc Dunn’s test was applied. The difference between AA and GG was shown for body mass (p=0.0211) and between AA and AG for glucose concentration (p=0.0444).

To investigate the differences between particular genotype carriers within specific groups, we compared anthropometric measurements and selected metabolic parameters for all genotypes in six subgroups: control and study group; the group with and without MetS; men and women. In the study group, there were no differences between TT, CT and CC carriers of rs2268641 when the following parameters were compared: age, body mass, BMI, WC, NC, and glucose, TG, HDL, AST and ALT concentration. Similarly, no statistically significant associations were found for the same parameters for the control group. A similar analysis was performed on the carriers of particular genotypes in the group with and without MetS. According to the results, there was only one statistically significant difference, where TT carriers had lower glucose concertation in comparison to CC carriers (97.69 mg/dl ± SD=23.27 and 107.34 mg/dl ± SD=27.97, respectively). This association was not found for the particular genotype carriers without MetS, however, in this group, TT carriers had lower NC (34.38 cm ± SD=3.14) when compared to CT (35.82 cm ± SD= 3.86; p = 0.0158) and CC carriers (35.85 cm ± SD=3.79; p = 0.0159). For this SNV, there were no differences between particular genotype carriers in men and women. Detailed data from the described analyses are available as **Supplementary Materials** (**Supplementary Tables 3–8**).

Similar analyses were performed for particular genotype carriers of the rs6923761 variant. The following parameters were compared: age, body mass, BMI, WC, NC, and glucose, TG, HDL, AST and ALT concentration. We found no differences between particular genotype carriers in the control and the study group, nor in the group with or without MetS. For this SNV, there were also no differences between particular genotype carriers in men and women. Detailed data from the described analyses are presented in **Supplementary Materials** (**Supplementary Tables 9–14**).

## Discussion

In this study, we found the association of two *GLP1R* SNVs, rs2268641 and rs6923761, with anthropometric measurements and metabolic parameters. No association of the studied SNVs with MetS was found. The disparity in the study and the control group count was expected and was most likely associated with the prevalence of excessive weight in Polish men and women (overweight prevalence 66% and 51%, respectively) (25). Similarly, the significantly different prevalence of MetS in both groups was expected, as central obesity is one of the criteria of MetS (24). In our population, the GG genotype of rs6923761 was associated with a lower risk, and A allele and AA genotype was associated with a higher risk of excessive body weight. This result is opposite to the observations made by de Luis et al. in their studies on the rs6923761 variant. According to their research work, the A allele carriers of the rs6923761 variant are characterised by lower values of anthropometric variables, including body mass and BMI (17, 18, 21, 26, 27). However, those results do not represent the general population, as all analyses were performed on obese subjects. Further research is needed to establish how particular genotypes of rs6923761 are associated with the risk of obesity and if those associations are different in specific subgroups, including the general population and subjects with excessive weight.

Our statistical analysis of the distribution of the particular genotypes of rs2268641 also showed significant differences between the study and the control group, as homozygous carriers of the minor allele had a lower risk of excessive body mass in our population. So far, only one study investigated the association of the rs2268641 variant with anthropometric attributes. The previous research work used a Bayesian hierarchical generalized linear model. In the analysed sample of New York European Americans, the rs2268641 variant was significantly associated with BMI (11). Further studies are needed to investigate the relationship of particular genetic variants of rs2268641 with excessive weight and anthropometric measurements.

Our study suggests that none of the studied SNVs is the risk factor for MetS. So far, only one study has investigated the relationship between the rs6923761 variant and MetS. De Luis et al. included 1122 obese participants in their analysis, where the prevalence of MetS was 47.4% and concluded that studied SNV was not a risk factor for MetS or its components (28). To our knowledge, no studies have examined the relationship between the rs2268641 variant and MetS to date. Further research is needed to confirm the lack of relationship of rs6923761 and rs2268641 with MetS.

This is the first study to investigate the association of the rs2268641 variant with various anthropometric measurements and metabolic parameters. We found no differences in the anthropometric and biochemical parameters between particular genotype carriers of the rs2268641 variant; however, the differences were identified when specific subgroups were investigated. In the MetS group, homozygous carriers of the minor allele had lower glucose concertation in comparison to homozygous carriers of the major allele. Another association was found in the group without MetS, where homozygous carriers of the minor allele lower NC than CT and CC carriers. These results suggest that the particular genotypes of this variant might be associated with anthropometric measurements and metabolic parameters, but only in specific subgroups. Future studies will help to establish potential mechanisms in which different genetic variants of rs2268641 may positively or negatively impact metabolic health.

We found significant differences between particular genotype carriers of rs6923761 for anthropometric and metabolic parameters. Homozygous carriers of the minor allele had higher body mass than GG carriers and higher glucose concentration than heterozygous subjects. Our results are opposite to the available research by de Luis et al., where the presence of the minor A allele was associated with lower BMI (17–19, 21, 28), body mass (17–19, 21, 28), WC (17–19, 21, 28), TG (17–19, 22), and glucose concentration (22). Moreover, in one of the studies, A allele carriers were characterised by higher HDL concentration (17). It needs to be noted that those studies were performed only on the obese population with various metabolic disorders; therefore, they cannot be directly compared to the results for the general population. We did not identify studies investigating the relationship of NC, ALT and AST with the rs6923761 variant.

The mechanism explaining how *GLP-1R* variants may be associated with body mass is not fully understood. One of the hypotheses is that SNV may be capturing the functional effects of a flanking-linked SNV, and variation of this locus may influence adipogenesis (29). Besides glucose homeostasis, GLP-1 is responsible for the reduction in food intake and appetite, increasing satiety and decreasing gastric emptying. It affects adipose cells, bone metabolism and the cardiovascular system (30). Research suggests that some SNVs might influence anthropometric measurements through satiety, appetite ratings and overeating, which was confirmed by a large number of reports for the *FTO* gene (31). Such extensive data is not available for rs2268641 and rs6923761 variants; however, de Luis et al. did not show any differences in energy intake or macronutrient distribution between alleles of rs6923761 (18). On the other hand, a study in mice found that genetic variation in *GLP1R* contributes to the differences in the gastric emptying rate and, ultimately, in food intake (32). Sathananthan et al. showed in the pilot study that rs6923761 is associated with altered β-cell responsivity in response to GLP-1 infusion. This variant results in the substitution of serine for glycine at position 168, and the authors concluded that GLP-1R variations might alter insulin secretion in response to exogenous GLP-1. However, this observation likely occurs only at supraphysiologic GLP-1 concentrations (33). On the contrary, Fortin et al. showed that ten missense variants, including rs6923761, exhibit normal basal and agonist-induced signalling (34). A study in naïve patients with T2DM demonstrated that a minor allele A of rs6923761 was associated with higher levels of basal GLP-1 (16). However, it should be noted that GLP-1 concentration may be altered in diabetic subjects, depending on the state of glucose control, duration of the disease and BMI status, therefore it is not possible to establish if the differences in GLP-1 concentrations are caused by *GLP1R* variation (30). So far, available data does not allow to conclude how rs6923761 and rs2268641 influence GLP-1 concentration and function, and if this could potentially explain the association of *GLP1R* variants with excessive body mass.

GLP-1 is one of the incretins that stimulates insulin secretion after oral glucose intake and is responsible for the regulation of glucose homeostasis (30). Therefore, the receptor of this hormone could be a candidate for regulating glucose concentration and mediating insulin secretion. Our results suggest that rs6923761 and rs2268641 are associated with fasting glucose; however, for rs2268641, this association was present only for the specific subgroup. Future studies should investigate this research question to establish the relationship between *GLP1R* variation and glucose homeostasis. Moreover, exploring the possible association of studied variants with T2D risk would contribute to a better understanding of the pathophysiology of this disease.

Glucagon-like peptide 1 receptor agonists (GLP-1RAs) are a class of medications used in the pharmacotherapy of obesity and T2D. Research shows that variation in *GLP1R* may be associated not only with the disease risk but also with the effectiveness of GLP1-RA treatment (35, 36). In two studies on Chinese subjects, carriers of specific genotypes of *GLP1R* variants were characterised by more pronounced weight loss and a bigger reduction in glycated haemoglobin (HbA1c) (37, 38). Research suggests that the pharmacological impact of *GLP1R* variants may be pathway and ligand-specific, where some variants may result in changes in certain signalling pathways (36). So far, the influence of rs2268641 on inter-individual GLP-1RA treatment effectiveness was not studied; however some data on the rs6923761 variant is available. A randomized clinical trial including otherwise healthy adults with obesity showed that this variant was associated with a reduction in percent body fat (39). Research on overweight and diabetic subjects also confirmed this association, as A allele carriers treated with GLP1-RA liraglutide had a greater decrease in weight and fat mass. The genetic variation was not associated with the parameters related to glucose homeostasis (29). On the contrary, the study investigating weight lowering potential of liraglutide in obese women with polycystic ovary syndrome (PCOS) did not confirm the better weight loss or glucose concentration outcomes for either allele of rs6923761 (40). Large-scale pharmacogenomic studies should investigate how genetic variation in *GLP1R* may influence GLP-1RA treatment effectiveness. A better understanding of how rs6923761 and rs2268641 variants influence disease risk and their successful treatment would significantly impact precision medicine development.

There are several limitations of our study. Due to the large sample size, single parameters and variables were not available for all participants (e.g. difficulties with blood collection, sample haemolysis). As a result, determination of MetS status was not possible for some subjects. Moreover, the genotype data was not available for the whole dataset, as a small part of the samples failed the genotyping and were excluded from further statistical analysis.

In summary, this is the first study investigating the association of rs2268641 and rs6923761 with excessive body mass, MetS, anthropometric measurements and metabolic parameters in the Polish population. In our study, AA carriers of rs6923761 had a higher risk of having excessive weight. Moreover, homozygous carriers of the minor allele had higher glucose concentration in comparison to AG carriers. Rs2268641 variant was also associated with body weight, as according to the results, TT carriers had a lower risk of excessive body mass. We did not find the association of rs2268641 and rs6923761with MetS.

Additional research in the general population is needed to explicitly establish the association of rs2268641 and rs6923761 *GLP1R* variants with MetS, anthropometric measurements and metabolic parameters. Further investigation of this research question could lead to the identification of potential mechanisms in which *GLP1R* variants influence body mass and metabolic health.

## Data availability statement

The datasets presented in this study can be found in online repositories. The names of the repository/repositories and accession number(s) can be found below: https://www.ebi.ac.uk/ena, PRJEB54891.

## Ethics statement

The studies involving human participants were reviewed and approved by Ethics Committee at Poznan University of Medical Sciences. The patients/participants provided their written informed consent to participate in this study.

## Author contributions

JM, AM, EM-K and PB conceived and designed the experiment. JM and AM performed genotyping. JM, EM-K and AS-J analysed the data. All authors contributed to the article and approved the submitted version.

## Funding

The study was funded by Poznan University of Medical Sciences from the internal funds of the Department of Treatment of Obesity, Metabolic Disorders and Clinical Dietetics.

## Conflict of interest

The authors declare that the research was conducted in the absence of any commercial or financial relationships that could be construed as a potential conflict of interest.

## Publisher’s note

All claims expressed in this article are solely those of the authors and do not necessarily represent those of their affiliated organizations, or those of the publisher, the editors and the reviewers. Any product that may be evaluated in this article, or claim that may be made by its manufacturer, is not guaranteed or endorsed by the publisher.
