# Supplementary material for: Association of GLP1R variants rs2268641 and rs6923761 with obesity and other metabolic parameters in a Polish cohort
Source: Front Endocrinol (Lausanne). 2022 Oct 19;13:1000185. doi: 10.3389/fendo.2022.1000185 (PMC9626533; doi:10.3389/fendo.2022.1000185)
Supplement: Supplementary file 1 [file Table_1.docx]

Supplementary Material

# Supplementary Tables

## Supplementary Table 1

HRM parameters and primer sequences for genotyping of rs226864 and rs6923761 variants.

| SNV | Chromosome position | Alleles | Primers for PCR amplification (5′–3′) | Annealing Temp. (°C) | Melt. Temp. Range (°C) |
| --- | --- | --- | --- | --- | --- |
| rs2268641 | chr6: 39082490 | C/T | Left: GGCAAGATCCCACTTAATGC  Right: GCTGGGTCCTCTAAGACCTG | 66 | 82–92 |
| rs6923761 | chr6: 39066296 | G/A | Left: CATCTACACGGTGGGCTACG  Right: GGGCCACCTTACCTGAAGC | 55 | 79–91 |

HRM, high-resolution melting curve analysis; SNV, Single Nucleotide Variant; PCR, polymerase chain reaction

## Supplementary Table 2

Comparison of anthropometric measurements and selected metabolic parameters between particular genotypes of rs2268641 variant.

| rs2268641 | TT | | CT | | CC | | p |
| --- | --- | --- | --- | --- | --- | --- | --- |
|  | Mean | SD | Mean | SD | Mean | SD |  |
| Age [years] | 52.63 | 14.76 | 53.71 | 13.96 | 54.48 | 14.40 | 0.4995 |
| Body weight [kg] | 73.34 | 15.64 | 75.60 | 15.14 | 75.22 | 15.39 | 0.1744 |
| BMI [kg/m^2^] | 26.63 | 5.07 | 27.30 | 4.92 | 26.93 | 4.93 | 0.1416 |
| WC [cm] | 90.79 | 15.15 | 93.05 | 14.35 | 92.34 | 14.41 | 0.3114 |
| NC [cm] | 35.70 | 3.71 | 36.55 | 3.81 | 36.55 | 3.83 | 0.0605 |
| Glucose [mg/dl] | 91.64 | 18.10 | 96.51 | 27.40 | 95.86 | 23.17 | 0.1529 |
| TG [mg/dl] | 149.47 | 99.84 | 151.71 | 117.41 | 149.26 | 105.02 | 0.9139 |
| HDL [mg/dl] | 65.31 | 17.29 | 64.07 | 17.20 | 64.74 | 16.53 | 0.7136 |
| AST | 29.10 | 14.95 | 28.53 | 11.45 | 28.28 | 10.26 | 0.5682 |
| ALT | 31.28 | 22.93 | 32.33 | 19.04 | 30.69 | 23.47 | 0.2276 |

SD, standard deviation; BMI, Body Mass Index; WC, waist circumference; NC, neck circumference; TG, triglycerides; HDL, high density lipoprotein; AST, aspartate transaminase; ALT, alanine transaminase. Kruskal-Wallis test was performed for this analysis.

## Supplementary Table 3

Comparison of anthropometric measurements and selected metabolic parameters between particular genotypes of rs2268641 variant in the study group.

| rs2268641 | Study group | | | | | | | |
| --- | --- | --- | --- | --- | --- | --- | --- | --- |
|  | TT | | CT | | CC | | p |  |
|  | Mean | SD | Mean | SD | Mean | SD |  |  |
| Age [years] | 58.44 | 11.63 | 56.47 | 13.20 | 57.26 | 13.68 | 0.4652 |  |
| Body mass [kg] | 82.54 | 13.78 | 81.95 | 13.40 | 82.73 | 13.24 | 0.7750 |  |
| BMI [kg/m^2^] | 29.97 | 4.29 | 29.74 | 4.07 | 29.60 | 4.06 | 0.7878 |  |
| WC [cm] | 100.44 | 11.55 | 98.98 | 12.33 | 99.51 | 11.65 | 0.5910 |  |
| NC [cm] | 37.55 | 3.25 | 37.56 | 3.65 | 37.72 | 3.45 | 0.8822 |  |
| Glucose [mg/dl] | 94.48 | 19.65 | 99.64 | 30.98 | 98.18 | 26.06 | 0.8359 |  |
| TG [mg/dl] | 185.36 | 113.16 | 171.86 | 130.38 | 165.58 | 112.96 | 0.5133 |  |
| HDL [mg/dl] | 58.44 | 14.61 | 60.39 | 15.27 | 61.23 | 15.16 | 0.2988 |  |
| AST | 32.07 | 18.41 | 29.21 | 12.60 | 29.22 | 11.01 | 0.8509 |  |
| ALT | 38.22 | 27.53 | 34.99 | 20.05 | 34.05 | 27.44 | 0.3840 |  |

SD, standard deviation; BMI, Body Mass Index; WC, waist circumference; NC, neck circumference; TG, triglycerides; HDL, high density lipoprotein; AST, aspartate transaminase; ALT, alanine transaminase; Kruskal-Wallis test was performed for this analysis.

## Supplementary Table 4

Comparison of anthropometric measurements and selected metabolic parameters between particular genotypes of rs2268641 variant in the control group.

| rs2268641 | Control group | | | | | | | |
| --- | --- | --- | --- | --- | --- | --- | --- | --- |
|  | TT | | CT | | CC | | p |  |
|  | Mean | SD | Mean | SD | Mean | SD |  |  |
| Age [years] | 45.55 | 15.31 | 48.53 | 14.19 | 49.79 | 14.52 | 0.1287 |  |
| Body mass [kg] | 61.70 | 8.47 | 62.80 | 9.16 | 62.06 | 8.53 | 0.6084 |  |
| BMI [kg/m^2^] | 22.40 | 1.71 | 22.37 | 1.83 | 22.24 | 1.86 | 0.8649 |  |
| WC [cm] | 78.49 | 9.19 | 80.88 | 9.82 | 79.40 | 8.75 | 0.2717 |  |
| NC [cm] | 33.03 | 2.55 | 33.92 | 2.82 | 34.25 | 3.50 | 0.0661 |  |
| Glucose [mg/dl] | 87.51 | 15.59 | 89.72 | 15.99 | 92.01 | 16.77 | 0.0760 |  |
| TG [mg/dl] | 96.65 | 46.49 | 110.87 | 65.64 | 124.05 | 84.40 | 0.2022 |  |
| HDL [mg/dl] | 75.56 | 15.90 | 72.36 | 18.75 | 71.14 | 17.34 | 0.1760 |  |
| AST | 24.60 | 5.40 | 27.07 | 7.56 | 26.52 | 8.51 | 0.1814 |  |
| ALT | 21.50 | 6.82 | 25.83 | 14.31 | 24.25 | 10.15 | 0.1800 |  |

SD, standard deviation; BMI, Body Mass Index; WC, waist circumference; NC, neck circumference; TG, triglycerides; HDL, high density lipoprotein; AST, aspartate transaminase; ALT, alanine transaminase; Kruskal-Wallis test was performed for this analysis.

## Supplementary Table 5

Comparison of anthropometric measurements and selected metabolic parameters between particular genotypes of rs2268641 variant in the group with metabolic syndrome.

| rs2268641 | MS criteria met | | | | | | |
| --- | --- | --- | --- | --- | --- | --- | --- |
|  | TT | | CT | | CC | | p |
|  | Mean | SD | Mean | SD | Mean | SD |  |
| Age [years] | 60.67 | 10.23 | 59.81 | 12.42 | 60.03 | 12.11 | 0.9803 |
| Body mass [kg] | 81.83 | 15.40 | 82.47 | 14.81 | 80.19 | 14.76 | 0.6057 |
| BMI [kg/m^2^] | 30.03 | 4.54 | 30.39 | 4.95 | 29.25 | 4.84 | 0.1315 |
| WC [cm] | 101.09 | 12.17 | 101.67 | 12.55 | 99.89 | 12.91 | 0.6893 |
| NC [cm] | 37.70 | 3.68 | 37.72 | 3.50 | 37.59 | 3.67 | 0.8955 |
| Glucose [mg/dl] | 97.69 | 23.27 | 109.91 | 38.04 | 107.34 | 27.97 | 0.0247 |
| TG [mg/dl] | 232.50 | 112.78 | 218.61 | 153.59 | 215.23 | 131.13 | 0.4551 |
| HDL [mg/dl] | 55.20 | 15.21 | 56.47 | 15.05 | 58.89 | 14.88 | 0.1556 |
| AST | 31.61 | 16.93 | 29.33 | 10.54 | 29.07 | 11.45 | 0.8113 |
| ALT | 38.61 | 24.13 | 35.68 | 19.38 | 32.86 | 18.41 | 0.3039 |

SD, standard deviation; BMI, Body Mass Index; WC, waist circumference; NC, neck circumference; TG, triglycerides; HDL, high density lipoprotein; AST, aspartate transaminase; ALT, alanine transaminase; Kruskal-Wallis test was performed and when p<0.05 post hoc Dunn’s test was applied. The difference between TT and CC was shown for glucose concentration (p=0.0247).

## Supplementary Table 6

Comparison of anthropometric measurements and selected metabolic parameters between particular genotypes of rs2268641 variant in the group without metabolic syndrome.

| rs2268641 | MS criteria not met | | | | | | |
| --- | --- | --- | --- | --- | --- | --- | --- |
|  | TT | | CT | | CC | | p |
|  | Mean | SD | Mean | SD | Mean | SD |  |
| Age [years] | 48.75 | 15.48 | 50.63 | 13.74 | 52.17 | 14.83 | 0.2093 |
| Body mass [kg] | 68.47 | 14.22 | 72.12 | 14.23 | 71.78 | 14.82 | 0.0697 |
| BMI [kg/m^2^] | 25.00 | 4.63 | 25.88 | 4.15 | 25.58 | 4.50 | 0.0833 |
| WC [cm] | 85.20 | 14.21 | 88.83 | 13.27 | 88.32 | 13.97 | 0.0841 |
| NC [cm] | 34.38 | 3.14 | 35.82 | 3.86 | 35.85 | 3.79 | 0.0102 |
| Glucose [mg/dl] | 88.10 | 13.22 | 87.88 | 12.17 | 88.50 | 16.25 | 0.9173 |
| TG [mg/dl] | 98.11 | 43.10 | 111.32 | 60.43 | 107.57 | 52.74 | 0.2662 |
| HDL [mg/dl] | 71.46 | 15.56 | 68.56 | 16.96 | 68.49 | 16.65 | 0.1679 |
| AST | 27.31 | 13.39 | 28.00 | 12.06 | 27.47 | 8.96 | 0.4581 |
| ALT | 26.68 | 21.17 | 30.13 | 18.59 | 28.59 | 25.00 | 0.0736 |

SD, standard deviation; BMI, Body Mass Index; WC, waist circumference; NC, neck circumference; TG, triglycerides; HDL, high density lipoprotein; AST, aspartate transaminase; ALT, alanine transaminase; Kruskal-Wallis test was performed and when p<0.05 post hoc Dunn’s test was applied. Differences between groups were shown for NC (TT vs CT p=0.0158; TT vs CC p=0.0159).

## Supplementary Table 7

Comparison of anthropometric measurements and selected metabolic parameters between particular genotypes of rs2268641 variant in women.

| rs2268641 | Women | | | | | | |
| --- | --- | --- | --- | --- | --- | --- | --- |
|  | TT | | CT | | CC | | p |
|  | Mean | SD | Mean | SD | Mean | SD |  |
| Age [years] | 52.54 | 14.93 | 53.52 | 14.76 | 53.97 | 14.68 | 0.7543 |
| Body mass [kg] | 70.29 | 14.92 | 70.61 | 14.23 | 70.64 | 14.89 | 0.8823 |
| BMI [kg/m^2^] | 26.43 | 5.35 | 27.11 | 5.48 | 26.88 | 5.39 | 0.3797 |
| WC [cm] | 88.58 | 15.46 | 89.49 | 14.52 | 89.22 | 14.80 | 0.8707 |
| NC [cm] | 34.68 | 3.12 | 34.74 | 2.89 | 34.83 | 3.11 | 0.8943 |
| Glucose [mg/dl] | 92.23 | 19.46 | 95.01 | 23.19 | 93.95 | 20.24 | 0.6573 |
| TG [mg/dl] | 142.12 | 92.54 | 133.8 | 71.41 | 141.9 | 107.37 | 0.9772 |
| HDL [mg/dl] | 67.89 | 17.21 | 69.00 | 16.55 | 68.48 | 17.15 | 0.9004 |
| AST | 29.17 | 16.06 | 27.23 | 8.73 | 27.26 | 9.08 | 0.9478 |
| ALT | 30.26 | 23.80 | 28.45 | 15.26 | 28.12 | 15.31 | 0.9614 |

SD, standard deviation; BMI, Body Mass Index; WC, waist circumference; NC, neck circumference; TG, triglycerides; HDL, high density lipoprotein; AST, aspartate transaminase; ALT, alanine transaminase; Kruskal-Wallis test was performed for this analysis.

## Supplementary Table 8

Comparison of anthropometric measurements and selected metabolic parameters between particular genotypes of rs2268641 variant in men.

| rs2268641 | Men | | | | | | |
| --- | --- | --- | --- | --- | --- | --- | --- |
|  | TT | | CT | | CC | | p |
|  | Mean | SD | Mean | SD | Mean | SD |  |
| Age [years] | 52.94 | 14.31 | 54.06 | 12.33 | 55.45 | 13.87 | 0.6040 |
| Body mass [kg] | 84.54 | 13.06 | 85.05 | 12.01 | 83.84 | 12.37 | 0.6926 |
| BMI [kg/m^2^] | 27.37 | 3.85 | 27.66 | 3.65 | 27.00 | 3.93 | 0.4176 |
| WC [cm] | 98.66 | 10.93 | 99.65 | 11.44 | 98.08 | 11.70 | 0.5469 |
| NC [cm] | 40.06 | 2.82 | 40.08 | 2.76 | 39.89 | 2.73 | 0.8517 |
| Glucose [mg/dl] | 89.24 | 10.92 | 99.52 | 34.22 | 99.80 | 27.99 | 0.0926 |
| TG [mg/dl] | 180.85 | 123.61 | 186.28 | 169.92 | 164.29 | 98.91 | 0.9058 |
| HDL [mg/dl] | 54.31 | 12.99 | 54.55 | 14.21 | 57.11 | 12.06 | 0.1239 |
| AST | 28.78 | 8.45 | 31.03 | 15.11 | 30.28 | 12.06 | 0.7089 |
| ALT | 35.91 | 18.25 | 39.77 | 23.00 | 35.75 | 33.82 | 0.0628 |

SD, standard deviation; BMI, Body Mass Index; WC, waist circumference; NC, neck circumference; TG, triglycerides; HDL, high density lipoprotein; AST, aspartate transaminase; ALT, alanine transaminase; Kruskal-Wallis test was performed for this analysis.

## Supplementary Table 9

Comparison of anthropometric measurements and selected metabolic parameters between particular genotypes of rs6923761 variant in the study group.

| rs6923761 | Study group | | | | | | |
| --- | --- | --- | --- | --- | --- | --- | --- |
|  | AA | | AG | | GG | | p |
|  | Mean | SD | Mean | SD | Mean | SD |  |
| Age [years] | 55.90 | 13.78 | 57.32 | 13.44 | 56.93 | 12.84 | 0.7110 |
| Body mass [kg] | 84.60 | 13.30 | 81.95 | 13.98 | 81.77 | 12.66 | 0.2054 |
| BMI [kg/m^2^] | 30.01 | 4.92 | 29.79 | 4.01 | 29.52 | 3.76 | 0.7712 |
| WC [cm] | 100.14 | 12.13 | 99.82 | 12.28 | 98.63 | 11.61 | 0.5751 |
| NC [cm] | 37.76 | 3.72 | 37.66 | 3.68 | 37.59 | 3.34 | 0.9979 |
| Glucose [mg/dl] | 100.47 | 28.65 | 98.60 | 31.56 | 97.79 | 24.03 | 0.3033 |
| TG [mg/dl] | 173.29 | 138.49 | 165.50 | 108.89 | 173.66 | 129.23 | 0.7568 |
| HDL [mg/dl] | 61.88 | 14.40 | 60.69 | 15.60 | 60.14 | 15.62 | 0.4769 |
| AST | 28.31 | 6.74 | 29.79 | 13.49 | 29.63 | 14.01 | 0.7477 |
| ALT | 31.71 | 13.89 | 35.78 | 27.63 | 35.20 | 21.63 | 0.7462 |

SD, standard deviation; BMI, Body Mass Index; WC, waist circumference; NC, neck circumference; TG, triglycerides; HDL, high density lipoprotein; AST, aspartate transaminase; ALT, alanine transaminase; Kruskal-Wallis test was performed for this analysis.

## Supplementary Table 10

Comparison of anthropometric measurements and selected metabolic parameters between particular genotypes of rs6923761 variant in the control group.

| rs6923761 | Control group | | | | | | |
| --- | --- | --- | --- | --- | --- | --- | --- |
|  | AA | | AG | | GG | | p |
|  | Mean | SD | Mean | SD | Mean | SD |  |
| Age [years] | 48.93 | 14.05 | 50.26 | 14.66 | 46.58 | 14.48 | 0.0806 |
| Body mass [kg] | 61.88 | 7.41 | 62.60 | 8.59 | 62.05 | 9.08 | 0.6732 |
| BMI [kg/m^2^] | 22.09 | 2.03 | 22.43 | 1.79 | 22.31 | 1.78 | 0.6920 |
| WC [cm] | 78.38 | 9.26 | 80.43 | 9.08 | 79.61 | 9.56 | 0.5133 |
| NC [cm] | 33.80 | 4.63 | 33.96 | 2.62 | 33.75 | 3.03 | 0.3576 |
| Glucose [mg/dl] | 96.77 | 17.46 | 89.29 | 15.29 | 89.86 | 17.15 | 0.0853 |
| TG [mg/dl] | 136.92 | 69.53 | 113.59 | 75.39 | 106.71 | 63.62 | 0.0832 |
| HDL [mg/dl] | 71.36 | 16.87 | 72.04 | 17.70 | 73.51 | 17.90 | 0.6588 |
| AST | 24.81 | 6.84 | 26.76 | 7.98 | 26.10 | 7.56 | 0.6505 |
| ALT | 22.57 | 8.43 | 24.21 | 11.56 | 24.73 | 12.28 | 0.8468 |

SD, standard deviation; BMI, Body Mass Index; WC, waist circumference; NC, neck circumference; TG, triglycerides; HDL, high density lipoprotein; AST, aspartate transaminase; ALT, alanine transaminase; Kruskal-Wallis test was performed for this analysis.

## Supplementary Table 11

Comparison of anthropometric measurements and selected metabolic parameters between particular genotypes of rs6923761 variant in the group with metabolic syndrome.

| rs6923761 | MS criteria met | | | | | | |
| --- | --- | --- | --- | --- | --- | --- | --- |
|  | AA | | AG | | GG | | p |
|  | Mean | SD | Mean | SD | Mean | SD |  |
| Age [years] | 59.51 | 12.67 | 60.86 | 11.69 | 59.48 | 12.15 | 0.6761 |
| Body mass [kg] | 84.99 | 14.55 | 80.56 | 15.25 | 81.49 | 14.51 | 0.1848 |
| BMI [kg/m^2^] | 30.94 | 5.78 | 29.83 | 4.85 | 29.63 | 4.39 | 0.5977 |
| WC [cm] | 102.87 | 13.05 | 100.54 | 13.15 | 100.91 | 11.95 | 0.6239 |
| NC [cm] | 37.99 | 4.08 | 37.62 | 3.69 | 37.73 | 3.40 | 0.8303 |
| Glucose [mg/dl] | 110.01 | 27.59 | 109.50 | 37.76 | 104.72 | 29.69 | 0.1362 |
| TG [mg/dl] | 220.49 | 156.91 | 218.74 | 125.36 | 215.53 | 147.70 | 0.6652 |
| HDL [mg/dl] | 59.38 | 14.80 | 57.31 | 14.59 | 56.42 | 15.85 | 0.2108 |
| AST | 27.95 | 7.65 | 30.71 | 13.14 | 28.83 | 11.78 | 0.2281 |
| ALT | 32.83 | 16.07 | 36.66 | 23.20 | 34.32 | 16.99 | 0.7469 |

SD, standard deviation; BMI, Body Mass Index; WC, waist circumference; NC, neck circumference; TG, triglycerides; HDL, high density lipoprotein; AST, aspartate transaminase; ALT, alanine transaminase; Kruskal-Wallis test was performed for this analysis.

## Supplementary Table 12

Comparison of anthropometric measurements and selected metabolic parameters between particular genotypes of rs6923761 variant in the group without metabolic syndrome.

| rs6923761 | MS criteria not met | | | | | | |
| --- | --- | --- | --- | --- | --- | --- | --- |
|  | AA | | AG | | GG | | p |
|  | Mean | SD | Mean | SD | Mean | SD |  |
| Age [years] | 50.72 | 14.02 | 52.17 | 14.64 | 49.44 | 14.22 | 0.1215 |
| Body mass [kg] | 73.04 | 14.36 | 72.27 | 14.90 | 69.95 | 13.80 | 0.2568 |
| BMI [kg/m^2^] | 25.68 | 4.00 | 25.96 | 4.37 | 25.32 | 4.38 | 0.2237 |
| WC [cm] | 88.83 | 14.20 | 89.58 | 13.88 | 86.26 | 13.17 | 0.0685 |
| NC [cm] | 35.85 | 4.41 | 35.86 | 3.71 | 35.23 | 3.59 | 0.2194 |
| Glucose [mg/dl] | 90.96 | 21.90 | 87.00 | 12.76 | 88.36 | 11.77 | 0.3095 |
| TG [mg/dl] | 117.68 | 57.75 | 104.67 | 49.69 | 107.19 | 59.30 | 0.2628 |
| HDL [mg/dl] | 68.02 | 15.35 | 68.49 | 17.15 | 70.30 | 16.75 | 0.2712 |
| AST | 27.02 | 6.07 | 27.64 | 11.37 | 27.73 | 12.27 | 0.5197 |
| ALT | 26.81 | 9.96 | 29.37 | 24.94 | 28.92 | 19.11 | 0.7791 |

SD, standard deviation; BMI, Body Mass Index; WC, waist circumference; NC, neck circumference; TG, triglycerides; HDL, high density lipoprotein; AST, aspartate transaminase; ALT, alanine transaminase; Kruskal-Wallis test was performed for this analysis.

## Supplementary Table 13

Comparison of anthropometric measurements and selected metabolic parameters between particular genotypes of rs6923761 variant in women.

| rs6923761 | Women | | | | | | |
| --- | --- | --- | --- | --- | --- | --- | --- |
|  | AA | | AG | | GG | | p |
|  | Mean | SD | Mean | SD | Mean | SD |  |
| Age [years] | 54.10 | 15.41 | 54.24 | 14.77 | 52.38 | 14.67 | 0.3052 |
| Body mass [kg] | 74.03 | 16.46 | 70.51 | 14.44 | 69.43 | 13.78 | 0.1283 |
| BMI [kg/m^2^] | 28.07 | 6.48 | 26.97 | 5.26 | 26.44 | 5.13 | 0.1177 |
| WC [cm] | 91.60 | 16.41 | 89.70 | 14.49 | 87.87 | 14.33 | 0.1970 |
| NC [cm] | 35.08 | 3.65 | 34.84 | 3.01 | 34.61 | 2.84 | 0.9035 |
| Glucose [mg/dl] | 97.88 | 23.30 | 93.86 | 23.51 | 93.48 | 19.32 | 0.1067 |
| TG [mg/dl] | 157.19 | 134.88 | 135.87 | 85.34 | 131.04 | 72.99 | 0.4112 |
| HDL [mg/dl] | 68.37 | 14.83 | 68.43 | 17.56 | 69.57 | 17.03 | 0.4337 |
| AST | 26.25 | 6.08 | 27.97 | 10.53 | 27.38 | 11.62 | 0.3246 |
| ALT | 27.52 | 11.81 | 29.11 | 17.72 | 28.18 | 17.83 | 0.5715 |

SD, standard deviation; BMI, Body Mass Index; WC, waist circumference; NC, neck circumference; TG, triglycerides; HDL, high density lipoprotein; AST, aspartate transaminase; ALT, alanine transaminase; Kruskal-Wallis test was performed for this analysis.

**1.14 Supplementary Table 14**

Comparison of anthropometric measurements and selected metabolic parameters between particular genotypes of rs6923761 variant in men.

| rs6923761 | Men | | | | | | |
| --- | --- | --- | --- | --- | --- | --- | --- |
|  | AA | | AG | | GG | | p |
|  | Mean | SD | Mean | SD | Mean | SD |  |
| Age [years] | 53.86 | 11.52 | 55.51 | 13.14 | 53.77 | 13.59 | 0.5451 |
| Body mass [kg] | 86.57 | 10.19 | 84.37 | 13.06 | 84.31 | 11.93 | 0.5241 |
| BMI [kg/m^2^] | 27.73 | 3.61 | 27.50 | 4.02 | 27.33 | 3.59 | 0.8144 |
| WC [cm] | 99.68 | 10.21 | 99.61 | 12.31 | 98.52 | 11.18 | 0.6166 |
| NC [cm] | 40.40 | 3.07 | 39.98 | 2.78 | 39.95 | 2.64 | 0.9756 |
| Glucose [mg/dl] | 102.28 | 30.10 | 98.77 | 34.02 | 97.82 | 26.85 | 0.5212 |
| TG [mg/dl] | 171.34 | 101.30 | 170.58 | 127.32 | 188.08 | 168.03 | 0.6609 |
| HDL [mg/dl] | 56.23 | 13.50 | 55.75 | 12.07 | 54.72 | 14.40 | 0.3338 |
| AST | 30.07 | 7.74 | 30.57 | 14.82 | 30.56 | 13.22 | 0.4898 |
| ALT | 33.76 | 15.36 | 38.40 | 33.65 | 38.55 | 20.94 | 0.3792 |

SD, standard deviation; BMI, Body Mass Index; WC, waist circumference; NC, neck circumference; TG, triglycerides; HDL, high density lipoprotein; AST, aspartate transaminase; ALT, alanine transaminase; Kruskal-Wallis test was performed for this analysis.
